# The economic impact of rostering junior doctors to triage to assist nursing staff in the early part of the patient journey through the emergency department

**DOI:** 10.1371/journal.pone.0261303

**Published:** 2021-12-17

**Authors:** David Brain, David Johnson, Julia Hocking, Angela T. Chang

**Affiliations:** 1 Australian Centre for Health Services Innovation (AusHSI) and Centre for Healthcare Transformation, School of Public Health & Social Work, Queensland University of Technology (QUT), Brisbane, Australia; 2 Hervey Bay Hospital Emergency Department, Wide Bay Hospital and Health Service, Queensland Health, Queensland, Australia; 3 Office for Research, Griffith University, Brisbane, Australia; 4 Centre for Allied Health Research, Royal Brisbane and Women’s Hospital, Brisbane, Australia; University of Malta Faculty of Health Sciences, MALTA

## Abstract

**Objective:**

This study aims to determine whether redeploying junior doctors to assist at triage represents good value for money and a good use of finite staffing resources.

**Methods:**

We undertook a cost-minimisation analysis to produce new evidence, from an economic perspective, about the costs associated with reallocating junior doctors in the emergency department. We built a decision-analytic model, using a mix of prospectively collected data, routinely collected administrative databases and hospital costings to furnish the model. To measure the impact of uncertainty on the model’s inputs and outputs, probabilistic sensitivity analysis was undertaken, using Monte Carlo simulation.

**Results:**

The mean costs for usual care were $27,035 (95% CI $27,016 to $27,054), while the mean costs for the new model of care were $25,474, (95% CI $25,453 to $25,494). As a result, the mean difference was -$1,561 (95% CI -$1,533 to -$1,588), with the new model of care being a less costly approach to managing staffing allocations, in comparison to the usual approach.

**Conclusion:**

Our study shows that redeploying a junior doctor from the fast-track area of the department to assist at triage provides a modest reduction in cost. Our findings give decision-makers who seek to maximise benefit from their finite budget, support to reallocate personnel within the ED.

## Introduction

Growing demand on Australia’s Emergency Departments (EDs) can lead to a reduction in the quality of patient care and is an issue faced all over the world [1–3]. Overcrowding is associated with patients waiting longer to be seen and treated, and this can lead to poorer health outcomes [4, 5]. Rostering consultant emergency physicians to work at ED triage has been shown to reduce waiting times, decrease total patient length of stay, decrease the number of patients leaving without complete assessment and decrease door-to-physician time in other jurisdictions [6]. However, this model of care is usually implemented by adding an emergency physician to the workforce, not reassigning them from elsewhere in the ED. This model is not feasible in EDs that have a shortage of senior staff or are unable to fund the substantial costs associated with the additional physician required.

This study aims to determine whether redeploying a junior doctor from the fast-track area of the department to assist at triage, front loading care and initiating early investigations and disposition where appropriate. Whether the implementation of this reallocation of existing staff provides good value for money in the Australian setting is not known. This paper presents an evaluation of the effectiveness of redeploying junior staff to triage, from an economic perspective in the ED of a regional public hospital in Queensland.

## Methods

This evaluation follows the guidelines presented in the *Consolidated Health Economic Evaluation Reporting Standards* checklist [7]. Following this checklist is recommended to ensure consistency and rigour when reporting economic findings. The completed checklist can be found in S1 Appendix. We undertook a cost-minimisation analysis–a suitable method of comparing costs of alternative approaches to care that are assumed to have an equivalent medical effect [8]. The study was approved by the Townsville Hospital and Health Service Human Research Ethics Committee, reference: HREC/17/QTHS/235_3.

### Description of intervention

The model was implemented in the ED of a regional public hospital in Queensland. At the time of the study the 12-bed department was seeing an average of 105 patients per day. During the study period, the usual number of rostered medical staff ranged between 10–13 junior doctors and 4 senior doctors over a 24-hour period. The new model of care consisted of rostering a junior doctor at the ED triage during the 10:00hrs– 20:00hrs shift. We defined a junior doctor as being a Principal House Officer or a Registrar, most commonly in year 3 or 4 of their postgraduate study. The doctor was rostered to triage and had the capacity to order investigations, stream patients to fast track, acute beds or the Clinical Decision Unit, and to treat and discharge patients with simple complaints. There was no change in the usual ED guidelines regarding supervision and senior approval for instigating investigations or discharging patients from the department. There was no restriction on the acuity level of patients assigned to the junior doctors who were rostered at triage. All patients were discussed with the senior medical officer, as per the ED policy, and if a patient was discharged directly from triage, their case was discussed with the senior doctor prior to being discharged.

### Usual care

On non-intervention days, junior doctors were in their usual role in the fast-track area of the ED.

### Study design

The study ran on consecutive days for a three-month period between February and May 2018. The new model of care was implemented every second day for the study period, (i.e.) Monday, Wednesday, Friday, Sunday in week one, followed by Tuesday, Thursday, Saturday in week two, and so on, for the study duration.

Data relating to ED length of stay (LOS), time of treatment commencement, investigations ordered, whether a patient did not wait for treatment or left before treatment commenced, and their destination—discharged home, admitted, transferred to another hospital or died; were collected via the Emergency Department Information System (EDIS). To estimate resource utilisation within the hospital, data from the hospital’s clinical costing unit was extracted for all patients who presented to ED in the study period.

Baseline demographic details for the study cohort are shown in Table 1. The groups were largely equivalent, with the number of patients and mean age being the same (45 years, SD = 26). There was a slightly higher percentage of females in the intervention group (51.4%) than the usual care group (50.2%).

**Table 1:** Demographic details of the study cohort.

	Usual Care	Intervention
n	2,784	2,784
Female, n (%)	1397 (50.2)	1431 (51.4)
Age, yrs: Mean (SD)	45 (26.8)	45 (26.8)
Avg. LOS in ED, minutes (SD)	242 (279)	239 (310)
**Patient Outcomes**	**n (%)**	**n (%)**
Admitted to hospital	315 (11.3)	312 (11.2)
Admitted to ED short stay	654 (23.5)	649 (23.3)
Admitted to ED	24 (0.9)	34 (1.2)
Discharged	1603 (57.6)	1642 (59.0)
Did not wait	100 (3.6)	81 (2.9)
Left after treatment commenced	55 (2.0)	33 (1.2)
Transfer to another hospital	31 (1.1)	31 (1.1)
Died in ED	2 (0.1%)	2 (0.1%)

### Description of decision-analytic model

**Fig 1:**
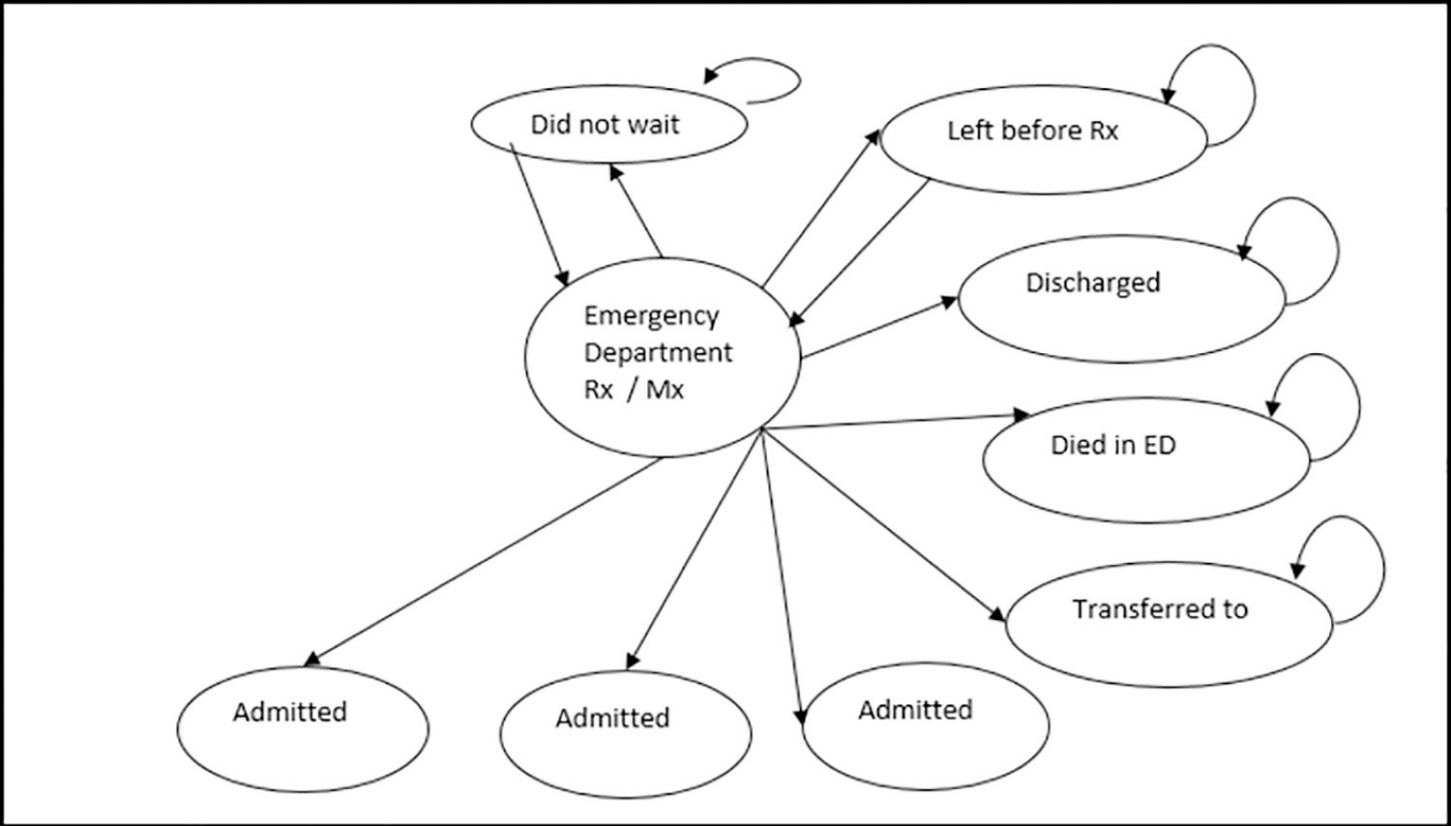
Pictorial representation of the decision-analytic model.

To evaluate the costs of the new model of care compared to usual care, a decision-analytic model was developed. To ensure that the model reflected the reality of ED accessing behaviour, it needed to be state-based and able to handle recursive events. As such, a Markov model was chosen, and a pictorial representation of the model is shown in Fig 1.

The model depicts the possible movement of patients through its states over time, with each state having a cost associated with time spent in that state. This structure provides the framework for the evaluation and is used to estimate costs associated with the differing approaches to service provision. The model contains nine states; ‘did not wait’, ‘ED treatment/Management’, ‘left before treatment commenced’, ‘discharged home’, ‘died in ED’, ‘transferred to another hospital’, ‘admitted to ED short stay’, ‘admitted to Hervey Bay Hospital (HBH)’ and ‘admitted to ED’. Given the short duration of visits to the ED, the model’s cycle length was hourly, and it was run for 24 cycles to complete a one-day time horizon. Due to the flexible nature of decision-analytic models such as this, extending the time horizon beyond one day is possible and easily achieved with some recalculation of the model’s input parameters. This may be useful for analysts who wish to use our model’s structure for analysis of outcomes in their jurisdiction.

All patients start in the ‘ED treatment/Management’ state. At each cycle of the model, patients in this state can either leave before treatment is commenced, be discharged home, not wait for treatment, be transferred to another hospital, be admitted to the ED’s short stay unit, be admitted as an inpatient, be admitted to ED, or die. When patients enter either the ‘discharged home’ or ‘transferred to another hospital’ states, they remain in that state for each cycle. Patients in the ‘left before treatment commenced’ or the ‘did not wait’ states can either remain in these states, or return to the model’s starting state, ‘ED treatment/Management’. This transition back to the ED represents a re-presentation within the one-day time horizon for the management of the same condition. Patients who are admitted–either to the ED, as inpatients at Hervey Bay hospital or to the ED short stay unit cannot transition elsewhere in the model. This reflects the fact that it is unlikely that the intervention will influence the management of patients after admission, so extension of the model beyond this point would not be an accurate representation of reality. The rate with which people transition through the states were estimated from prospectively collected administration data and are different according to whether a junior doctor was at triage (intervention) or fulfilling their usual role in the fast track area of the ED (usual care). The model was constructed and analysed in Microsoft Excel 2010.

### Input data for the model

Movement between health states is based on transition probabilities, which were estimated from various sources, while costs were derived from routinely collected administrative databases. These sources are shown in Table 2. The cost associated with being in each state were calculated per cycle and then summed over the 24 cycles of the model. Given that the model’s time horizon was less than 1 year, discounting was not applied [9].

**Table 2:** Input variables for the economic model.

Variable	Fixed Value (α, β)	Distribution	Source
**Transition Probabilities**			
Usual Care			
tpED Rx Mx_DNW	0.0093 (25, 2756)	Beta	Study Cohort
tpED Rx Mx_L	0.0021 (6, 2776)	Beta	Study Cohort
tpED Rx Mx_Die	0	Beta	Study Cohort
tpED Rx Mx_T	0.0003 (1, 2781)	Beta	Study Cohort
tpED Rx Mx_EDSS	0.0143 (40, 2742)	Beta	Study Cohort
tpED Rx Mx_ED	0	Beta	Study Cohort
tpED Rx Mx_HBH	0.0007 (2, 2780)	Beta	Study Cohort
tpED Rx Mx_Dis	0.0689 (192, 2590)	Beta	Study Cohort
tpDNW_ED Rx Mx^^^	0.08 (8, 92)	Beta	Study Cohort
tpL_ED Rx Mx^^^	0.1454 (8, 47)	Beta	Study Cohort
**Intervention**			
tpED Rx Mx_DNW	0.0082 (23, 2759)	Beta	Study Cohort
tpED Rx Mx_L	0.0021 (6, 2776)	Beta	Study Cohort
tpED Rx Mx_Die	0	Beta	Study Cohort
tpED Rx Mx_T	0.0003 (1, 2781)	Beta	Study Cohort
tpED Rx Mx_EDSS	0.020 (57, 2725)	Beta	Study Cohort
tpED Rx Mx_ED	0.0010 (3, 2779)	Beta	Study Cohort
tpED Rx Mx_HBH	0.0021 (6, 2776)	Beta	Study Cohort
tpED Rx Mx_Dis	0.1009 (281, 2501)	Beta	Study Cohort
tpDNW_ED Rx Mx^^^	0.1728 (14, 67)	Beta	Study Cohort
tpL_ED Rx Mx^^^	0.0909 (3, 30)	Beta	Study Cohort
**Costs**	**$ /Hour**	**Distribution**	**Source**
Usual Care	$137.70	Gamma	Clinical Costing Unit
Intervention	$143.47	Gamma	Clinical Costing Unit

Mx = Management; Rx = Treatment

### Transition probabilities

To estimate transition probabilities for the model, we used prospectively collected data from the study period. For the transition from ‘ED treatment / Management’ to each alternative state—‘did not wait’, ‘left before Rx commenced’, ‘died in ED’, ‘transferred to other hospital’, ‘admitted to EDSS’, ‘admitted to ED’, ‘admitted to HBH’ and ‘discharged home’—cumulative risk curves were calculated. The probability estimates for each hour over the 24-hour model period was calculated (R Studio, Version 1.2). The alpha and beta of these probability estimates were included in the model to calculate the probabilistic mean (beta distribution) for each transition probability. To estimate the probability of transitioning from the ‘did not wait’ and ‘left before treatment commenced’ states to ‘ED treatment / Management’, we identified the first presentation to the ED following their ‘did not wait’ or ‘left before treatment commenced’ episode. Clinical information regarding the presentation was reviewed by the project’s clinical lead to determine whether the presentation was a re-presentation for the management of the same condition, to ensure that the model included patients who came back to ED for the same reason as their initial presentation. The inclusion of patients who bounced back to the ED due to their original condition not being managed during their initial presentation or due to deterioration after discharge provides a more comprehensive model as it accounts for a small, but clinically important group. The number of events and number at risk were used to calculate the transition probability, using a beta distribution. As the model was 24 hours in duration, this probability was included as occurring in hour 24 of the model. The probability of remaining in a state was simply calculated as 1 minus the sum of the other probabilities associated with leaving that state, as per recommended methods [10]. Sources of evidence, and values used in the model are summarised in Table 2.

### Costs

Costs associated with each state were measured and valued in 2018 Australian dollars. The cost for each presentation to the ED over the study period was extracted from hospital costing by Urgency Related Groups classification version 1.3 [11]. These are inclusive of all direct and indirect cost components, including staffing, medications, investigations, imaging, consumables and overheads. The total cost and length of stay in minutes for all presentations managed in the intervention and usual care groups over the study period was calculated and an average cost per hour under each model was then derived. Using this approach, the average cost per hour for usual care was $137.70 and the average cost per hour for the intervention was $143.47. An estimated hourly cost for an additional junior medical officer ($69.09 for L4 / PHO1 / Registrar 1) was calculated from published wage rates and an additional 30% on-costs added to cover costs such as leave entitlements, superannuation and overheads [12].

The perspective chosen for this evaluation is the hospital perspective [13]. This means that we only included costs borne by the hospital, such as those associated with the administration, monitoring and in-hospital treatment of a patient’s condition. It does not include downstream costs, such as follow-up visits in primary care, or a patient’s out of pocket expenses, such as those associated with over the counter purchases co-payments, transport, parking or lost productivity, because these costs are beyond the scope of interest for this decision problem.

### Model outputs

The main purpose of this study is to understand the best utilisation of a limited staffing budget. Taking a cost-minimisation approach, we can compare the costs associated with usual care and those associated with the new model of care, to give decision-makers an understanding of how a simple rearrangement of available staff may impact their budget.

### Handling uncertainty

To measure the impact of uncertainty on the model’s inputs and outputs, probabilistic sensitivity analysis was undertaken. One thousand iterations of the model were completed, using Monte Carlo simulation, with each simulation taking a random draw from each parameter’s distribution [14]. Transition probabilities were assigned beta distributions, while costs were assigned gamma distributions, reflecting the skew that is associated with this type of data [14]. The change to costs was recorded for each model simulation, producing 1,000 estimations of the change. Interpreting whether the intervention is good value for money in comparison to usual care becomes straightforward–the model of care with the largest number of model simulations showing a cost-saving, becomes the model of care to adopt in practice.

Uncertainty in other aspects of the evaluation also exist and were explored through multiple scenario analyses. Scenario analysis is a useful approach to test the model’s robustness, but to also increase the breadth of information available to decision-makers about this decision problem. Key parameter values were changed in the model to reflect plausible scenarios in the Australian setting. Four alternate scenarios were considered: (1) when there were 3 doctors present at the ED, in addition to the junior doctor at triage; (2) when there were 2 doctors present at the ED, in addition to the junior doctor at triage; (3) when there was 1 doctor present at the ED, in addition to the junior doctor at triage; and (4) when an extra junior doctor was included on the roster, so that there was one junior doctor at triage, and one junior doctor in their usual role in the fast track area of ED. These scenarios were chosen as they represent the reality of staffing level variation in the ED, which is a common issue, particularly in small to medium sized departments. The results of these scenario analyses will provide decision-makers with information about whether there is any benefit implementing the new model when there are less than the ideal number of staff available on the roster.

## Results

All results are based on a model that ran for a time horizon of one day, with 102 patients–reflective of the average throughput of patients from the study period. The mean costs for usual care were $27,035 (95% CI$27,016 to $27,054). The mean costs for the new model of care were $25,474, (95% CI $25,453 to $25,494). As a result, the mean difference was -$1,561 (95% CI -$1,533 to -$1,588). Based on our model, this equates to a reduction in cost of approximately $16 per patient, per day. Cost-savings were driven by the reduction in LOS associated with the new approach to staff management. The overall probability that the new model of care was cost-saving, was 100%, given that all of the model’s 1,000 simulations resulted in a cost-reduction when a junior doctor was at triage, compared to their role in usual care. Table 3 shows the results of the base case analysis, as well as the scenario analyses.

**Table 3:** Results of the base case analysis and scenario analyses.

Model	Mean change (Min:Max)	Optimal Strategy	Probability cost-saving
Baseline	-$1,561 (-$175 to -$3,411)	Intervention	100%
Scenario 1: 3 Drs. rostered	-$2,195 (-$104 to -$4,478)	Intervention	100%
Scenario 2: 2 Drs. rostered	-$151 ($1,772 to -$2,311)	Intervention	57.5%
Scenario 3: 1 Dr. rostered	-$309 ($3,253 to -$3,344)	Intervention	60.5%
Scenario 4: Pay for extra Jr Dr.	-$1,546 (-$110 to -$2,853)	Intervention	100%

### Scenario analysis

Findings in Table 3 show that the new model of care is the optimal approach for all considered scenarios, although the probability that the new model of care is cost-saving does differ in some circumstances. For example, the results show a 100% probability that the new model of care is cost-saving when 3 doctors are rostered in addition to the junior doctor stationed at triage, but this probability is reduced when less than 3 doctors are rostered in addition to the junior doctor. The certainty in the result is reduced for scenarios 2 and 3, meaning that the new model of care may not be an obvious choice when the department is faced with less than the ideal number of doctors on the roster. However, the new model of care has a 100% probability of remaining cost-saving, even when an extra junior doctor is added to the rostered team. This suggests that the savings observed from having the junior doctor at triage are large enough to account for the extra cost of having another junior doctor rostered.

## Discussion

This study suggests that stationing a junior doctor at triage has a high probability of cost reduction when compared to utilising junior doctors in the fast track area of ED. These conclusions were consistent despite alterations to the model’s key parameters, and provide support for a simple, easy to implement rearrangement of the workforce, that could be applied in any ED that has junior doctors. The most common issue facing hospital-based decision-makers is that they are tasked with managing a constrained budget, but high expectations associated with patient care and clinical outcomes. Interestingly, our findings show that the use of junior doctors was still a cost-saving approach, even if that junior doctor was rostered in addition to usual staffing numbers.

There is also an implementation challenge associated with changing the way that staff are allocated at the emergency department. To ensure there is a smooth transition of these findings into practice, ED staff will need to be involved in the process of transition, likely requiring buy-in to training relating to the new approach. There is no shortage of examples where new innovations have been unsuccessful in the translation phase and decision-makers must be aware of the challenge and equipped to manage it [15]. At a minimum, clear leadership and support for staff associated with the implementation of the new approach is vital to its success.

### Comparison to existing literature

To our knowledge, this is the first study to evaluate a novel approach to the use of scarce budgetary resources for the medical workforce in Australia’s emergency departments. Our results mirror those from other jurisdictions such as the USA and UK, where the utilisation of emergency physicians at triage can reduce waiting times and decrease total ED length of stay [16, 17]. Further, a study from the USA [18] determined that the use of medical staff at triage was cost-effective and contributed to gains in patient satisfaction and a reduction in the proportion of people who left before treatment commenced. Whilst we did not measure patient satisfaction, we believe our results complement their assertion that the use of medical staff at triage can be good value for money. We believe that our findings are important and are a valid addition to the existing literature, as they incorporate key contextual factors associated with the Australian health system, which cannot be extrapolated from an American study.

## Limitations

Our study has limitations. First, the new model of care was only applied to one shift in the day, meaning the results primarily relate to this time period. Whether the new model of care achieves the same results at other times of the day is unknown and yet to be tested. Second, the scenario analysis was undertaken using a subsection of the data from intervention days with variable number of junior doctors. This was representative of the challenges in maintaining consistent staffing levels within an ED and provided an opportunity to undertake sensitivity analysis of the varying staffing levels. However, as the number of junior doctors was not controlled, there is a variation in the number of days / patients in each scenario resulting in lower number for Scenario 3 (one junior doctor) compared to Scenario 1 (three junior doctors) which led to an increase in variance of the parameters. Further, the study was conducted at one site in regional Queensland, and as such, care when generalising the results to other jurisdictions is advised. However, the arrangement of emergency departments is reasonably similar Australia-wide, so we are confident that our findings would be similar to those experienced elsewhere in the country. We have not considered implementation costs associated with the new model of care, which may have some impact on the results. Costs associated with staff education, training and education materials would be increased if a thorough implementation of the new approach to staffing was taken on at the hospital and would ideally be included in any evaluation from an economic perspective. Finally, this study did not determine whether the quality of care differed according to the group with/without a junior doctor at triage. This study focused on more efficient use of hospital staff at the ED, and further studies regarding how this impacts quality of care would be of benefit.

## Conclusion

Efficient deployment and utilisation of junior doctors within the ED is a constant juggle for ED directors and decision-makers. Our study shows that redeploying a junior doctor from the fast-track area of the department to assist at triage provides a modest reduction in cost. As a result, our findings give decision-makers who seek to maximise benefit from their finite budget, support to reallocate personnel within the ED. To our knowledge, our findings are novel in the Australian setting, and could be used to support further research on this topic in other Australian jurisdictions.

## Supporting information

S1 Appendix CHEERS checklist–items to include when reporting economic evaluations of health interventions. (DOCX)
